# Protection From Influenza by Intramuscular Gene Vector Delivery of a Broadly Neutralizing Nanobody Does Not Depend on Antibody Dependent Cellular Cytotoxicity

**DOI:** 10.3389/fimmu.2020.00627

**Published:** 2020-05-29

**Authors:** Joanne Marie M. Del Rosario, Matthew Smith, Kam Zaki, Paul Risley, Nigel Temperton, Othmar G. Engelhardt, Mary Collins, Yasuhiro Takeuchi, Simon E. Hufton

**Affiliations:** ^1^Division of Biotherapeutics, National Institute for Biological Standards and Control, Potters Bar, United Kingdom; ^2^Division of Advanced Therapies, National Institute for Biological Standards and Control, Potters Bar, United Kingdom; ^3^Division of Infection and Immunity, University College London, London, United Kingdom; ^4^Department of Physical Sciences and Mathematics, College of Arts and Sciences, University of the Philippines Manila, Manila, Philippines; ^5^Division of Virology, National Institute for Biological Standards and Control, Potters Bar, United Kingdom; ^6^Viral Pseudotype Unit, Medway School of Pharmacy, The Universities of Kent and Greenwich at Medway, Chatham, United Kingdom; ^7^Okinawa Institute of Science and Technology Graduate University, Okinawa, Japan

**Keywords:** influenza, vaccine, adeno associated viral vectors, immunoprophylaxis, immunotherapy, nanobody, monoclonal antibody, antibody dependent cellular cytotoxicity

## Abstract

Cross-subtype neutralizing single domain antibodies against influenza present new opportunities for immunoprophylaxis and pandemic preparedness. Their simple modular structure and single open reading frame format are highly amenable to gene therapy-mediated delivery. We have previously described R1a-B6, an alpaca-derived single domain antibody (nanobody), that is capable of potent cross-subtype neutralization *in vitro* of H1N1, H5N1, H2N2, and H9N2 influenza viruses, through binding to a highly conserved epitope in the influenza hemagglutinin stem region. To evaluate the potential of R1a-B6 for immunoprophylaxis, we have reformatted it as an Fc fusion for adeno-associated viral (AAV) vector delivery. Our findings demonstrate that a single intramuscular injection in mice of AAV encoding R1a-B6 fused to Fc fragments of different isotypes equipped either, with or without antibody dependent cellular cytotoxicity (ADCC) activity, was able to drive sustained high-level expression (0.5–1.1 mg/mL) in sera with no evidence of reduction for up to 6 months. R1a-B6-Fc fusions of both isotypes gave complete protection against lethal challenge with both pandemic A/California/07/2009 (H1N1)pdm09 and avian influenza A/Vietnam/1194/2004 (H5N1). This data suggests that R1a-B6 is capable of cross-subtype protection and ADCC was not essential for R1a-B6 efficacy. Our findings demonstrate AAV delivery of cross-subtype neutralizing nanobodies may be an effective strategy to prevent influenza infection and provide long-term protection independent of a host induced immune response.

## Introduction

Influenza virus continues to be a major public health concern, causing both annual epidemics and occasional pandemics (1). In a pandemic, a new influenza virus emerges and infects the human population which has little or no pre-existing immunity (2, 3). Vaccines remain the main method of infection control, however their timely implementation and poor immunogenicity in certain vulnerable patient groups remain a considerable problem (4). Although antiviral drugs such as Oseltamivir are available to control the spread of the virus their effectiveness is limited in treating patients with influenza (5, 6). There is clearly an urgent need for additional approaches and antibodies present new opportunities for both therapeutic and prophylactic intervention. Passive transfer of serum antibodies from convalescent patients has been used in the past (7, 8), however, this approach is of limited use in a global pandemic emergency. A much more promising strategy is to use recombinant monoclonal antibodies (mAbs) against influenza and several are currently in clinical development (9–13). These rare mAbs bind to functionally conserved epitopes such as those in the hemagglutinin (HA) stem, thereby providing strain independent protection. However, for passive immunotherapy to provide sufficient long-term protection, frequent repeated injections are required which would be prohibitively expensive in low resource areas. A more practical and cost-effective strategy would be to use antibody gene therapy which would provide long term sustainable protection through antibody production within the patient.

Viruses have been exploited as gene delivery vectors for many years owing to their highly evolved mechanisms for efficient delivery of genetic material to host cells (14). Recombinant Adeno-Associated virus (rAAV) vectors have been modified to improve safety and are suitable vectors for clinical gene therapy (15). The ability of rAAV vectors to provide long term stable transgene expression in different animal cells, their low immunogenicity, and overall versatility, make them the vector of choice for *in vivo* gene therapy (16–19). AAV-mediated delivery of broadly neutralizing human monoclonal antibodies against the HA stem has already been shown as a viable approach to protect from influenza (20, 21). The intramuscular injection of AAV8 expressing the cross-subtype neutralizing human mAb F10 could protect young, old, and immunocompromised mice from influenza challenge through sustained expression in the systemic circulation for at least 11 months at levels between 150 and 200 μg/mL (20). Similar studies have investigated the AAV-mediated delivery of another broadly neutralizing human mAb, FI6, which was shown to protect mice and ferrets from lethal influenza challenge. In this study FI6 was delivered intranasally which may be beneficial as this is the natural site of influenza infection (22, 23). Despite these findings, significant challenges remain for the successful development of vectored immunoprophylaxis for influenza. Although AAV is an excellent vector for gene therapy, it is still hampered by limitations to the size and complexity of antibody transgenes that it can express (20). This is a challenge for antibody gene therapy given that mAbs are large complex glycoproteins comprising four separate chains. As such, smaller, simpler binding molecules expressed from a single open reading frame would be a significant advantage (19, 21).

Structural analysis of several of the earliest human mAbs against the influenza HA stem revealed the unusual feature that they employ only their heavy chains for antigen recognition (10, 13). This implies that the light chains were not required for binding to these difficult to access epitopes. In addition, some of the most potent cross-neutralizing human mAbs described have very low levels of somatic hypermutation and are often constrained to particular germline genes (10, 11, 13, 24, 25). This suggests that they may be products of an immediate and sub-optimal immune response to influenza (26, 27). This prompted our interest in naturally occurring “heavy-chain only” antibodies from camelids and our isolation of high affinity broadly neutralizing single domain antibodies (nanobodies) against influenza A and B (28, 29). This antibody format is unique to camelid species (30) and can be isolated from immunized alpacas as highly optimized single domain binding units that have gone through extensive somatic hypermutation possibly due to the alpaca’s limited immune history of exposure to influenza (31). Nanobodies have several well-described advantages over conventional mAbs which make them ideal for applications in infectious disease (32–34). One interesting feature is that they have a preference for binding to clefts and grooves through unusually long CDR loops (35). In addition, their simple modular structure and single gene format enables easy engineering for different delivery and therapeutic applications (14, 28, 36–38). This next generation of antibodies has reached a significant milestone with the approval in September 2018 of the first nanobody, Caplicizumab^TM^, for the treatment of a blood clotting disorder (39).

We have previously described R1a-B6 as a potent alpaca derived nanobody capable of cross subtype neutralization of pandemic A(H1N1)2009, highly pathogenic avian influenza H5N1, H2N2 and H9N2 (28, 40). R1a-B6 neutralizes influenza through binding to a highly conserved epitope in the HA stem and blocking the low pH induced conformational change required for viral membrane fusion. Within this study we have evaluated if R1a-B6’s potent *in vitro* neutralizing activity can translate into *in vivo* efficacy. As a single domain antibody fragment of approximately 15 kDa, R1a-B6 would be rapidly cleared from circulation in a matter of minutes, which would prohibit any activity *in vivo* (41, 42). To achieve maximum protective levels in systemic circulation, additional strategies to enhance its pharmacokinetics are required, (43) such as fusion to an antibody Fc domain. The Fc domain is largely responsible for the extended serum persistence of mAbs by pH dependent interaction with FcRn and recycling through the kidneys (44). In addition, the Fc domain mediates interactions with the effector arm of the immune system and recent reports have suggested antibody dependent cellular cytotoxicity (ADCC) is required for efficacy of human mAbs specific for the influenza HA stem (45, 46).

Recent studies by Laursen and colleagues have also described cross-neutralizing single domain antibodies to influenza and furthermore shown *in vivo* efficacy by intranasal AAV delivery at the natural site of infection (47). In addition, they describe linking multiple single domain antibodies together specific to different epitopes on HA to provide almost complete protection against both influenza A and B virus in mouse challenge models. Our study confirms single domain antibodies can be delivered by gene therapy but is distinct in that we explore AAV8 intramuscular rather than AAV9 intranasal delivery of R1a-B6 in different formats to evaluate the relative importance of the Fc domain for prophylactic efficacy (47). Direct intramuscular injection of AAV8 allows for a simple method for crossing the blood vessel barrier to achieve gene transfer into muscle cells (48, 49). Muscle then becomes a biological factory for expressing antibodies *in vivo* and has previously been shown to be effective in mice against HIV (50) and influenza (20). AAV8 and AAV9 share the same tropism, however, low levels of protein expression and genome copy numbers of AAV9 has been detected in the brain and testes, which is an important safety issue for clinical development of this serotype (51). Our choice of muscle specific delivery and AAV8 as vector serotype is expected to provide higher level, more sustainable transgene expression over a longer period of time than AAV9 mediated intranasal delivery which has previously been shown to decrease in macaques after 3-4 months (22, 52, 53).

We report the relative prophylactic efficacy of R1a-B6 delivered in different formats designed to investigate the importance of the Fc domain for half-life extension and effector function. Our findings are discussed in the context of designing the optimum transgene for AAV vector delivery of R1a-B6 to accomplish long term, safe, broad protection from pandemic influenza in vulnerable patient groups.

## Materials and Methods

### Nanobody-Fc Expression and Purification

Nanobodies R1a-B6 (28) and a control anti-lysozyme specific nanobody cAb1 (54) sequences were reformatted as Fc fusions separated by a mouse hinge region. The mouse CH1 domain was removed and the C-terminal end of the nanobody was directly fused to the N-terminus of the mouse hinge region followed by the CH2-CH3 domains of either mouse IgG2a or mouse IgG1. All constructs were synthesized (IDT technologies) and cloned into pcDNA 3.4-TOPO (Invitrogen A14697) for mammalian expression. Large-scale preparation of endotoxin-free plasmid DNA using NucleoBond Xtra Midi EF Kit (Macherey Nagel 740420.10) was carried out. We then used the ExpiCHO Expression System (Thermo Fisher Scientific A21933) to produce soluble nanobody-Fc fusion proteins. Cell supernatants were purified using HiTrap Protein A HP (GE Healthcare 17040203) chromatography. Eluted fractions with high A280 readings that appeared as bands in the SDS-PAGE gels were pooled and dialyzed in a 10 L solution of 1X PBS overnight at 4°C.

### *In vitro* ADCC Reporter Assay

White opaque 96-well Nunclon (Thermo Scientific 165306) plates were coated overnight at 4°C with 5 μg/mL A/California/07/2009(H1N1) NYMC X-179A (NIBSC 09/174). Sample dilutions of nanobody-Fc ranging from 10 μg/mL to 0.25 ng/mL were made and ADCC Reporter Bioassay was then carried out as per manufacturer’s instructions (Promega M1201) and read using the GloMax Navigator (Promega).

### Specificity of Nanobody-Fc via ELISA

The nanobody-Fc constructs were tested against influenza antigen standards from the National Institute for Biological Standards and Control (NIBSC) as listed in Supplementary Table 3. Plates were coated overnight with 50 μL/well of 5 μg/mL influenza virus antigen standard diluted in PBS at 4°C. Serial two-fold dilutions ranging from 4 μg/mL to 1 ng/mL of purified R1a-B6-mIgG1, R1a-B6-mIgG2a, R1a-B6, and cAb1-mIgG2a were then added. Secondary antibody, anti-mouse IgG (Fc Specific)-Peroxidase (Sigma A0168) at a 1:2000 dilution was used to detect nanobody-Fc; while monoclonal anti-polyHistidine-Peroxidase clone HIS-1 (Sigma A7058) at a 1:1000 dilution was used to detect R1a-B6. TMB substrate (Thermo Scientific 34029) was added to the plate and the reaction was stopped with 0.5 M HCl or 0.5 M H_2_SO4. Plates were read on a SpectraMax M5 ELISA plate reader using SoftMax Pro software. Absorbance at 450 nm was taken.

### Pseudotype Neutralization Assay

The following pseudotypes with luciferase reporters (55), A/Korea/426/68(H2N2) (KR/68), A/Vietnam/1194/2004(H5N1) (VN/04), and A/Hong Kong/1073/99(H9N2) (HK/99) were used. Serial two-fold dilutions of nanobody-Fc were made ranging from 32 nM to 1 pM. Influenza pseudotypes at a concentration of 1.0 × 10^6^ RLU/well were then added to the nanobody-Fc dilutions and 1.0 × 10^4^ 293T cells were added to each well. Plates were incubated for 48 h at 37°C and 5% CO_2_. Supernatant was then harvested, and plates were read using the GloMax Navigator (Promega) using the Promega GloMax Luminescence Quick-Read protocol.

### Production of rAAV-Nanobody Constructs

AAV-R1a-B6-mIgG1, AAV-R1a-B6-mIgG2a, AAV-R1a-B6, and AAV-cAb1-mIgG2a, were generated by cloning the nanobody constructs into the AAV2 ITR-containing plasmid pTRUF11. The rAAV plasmids were transformed in XL-1 Blue (Stratagene 200249) competent cells via heat shock and scaled up in suspension cultures for large-scale DNA extraction using the NucleoBond Xtra Midi Endotoxin Free Kit (Macherey Nagel 740420.10). To check for ITR integrity, plasmid DNA was digested with *Sma*I (Promega) and run on a 1% (w/v) agarose gel.

### rAAV Production

HEK293T cells were grown to a density of 4.0 × 10^7^ 293T cells in a final volume of 100 mL per triple flask of Dulbecco’s Modified Essential Medium (DMEM) (Sigma D6546) supplemented with 10% (v/v) Fetal Calf Serum (FCS) (Sigma 14A138), 1% (v/v) L-glutamine (Sigma G7513), 1% (v/v) Penicillin-Streptomycin (PenStrep) (Sigma P0781). Twelve triple flasks (TF) were used per rAAV construct to make a cell factory. Recombinant AAV vectors pseudotyped with serotype eight capsid containing AAV2 ITRs (AAV2/8) were generated by transient transfection using polyethyleneimine (PEI) (PolySciences 24765-1) of pHelper, pAAV construct, and pAAV2/8/Rep-Cap. Flasks were incubated at 37°C and 5% CO_2_. After 72 h, virus was harvested and extracted from the supernatant via ammonium sulfate (NH_4_)_2_SO_4_ (Sigma A4418) precipitation. Cells in the triple flask were trypsinized and combined with the supernatant and subjected to three freeze/thaw cycles and treated with 10,000 U Benzonase (Novagen 70746-4). Tubes were then centrifuged to clarify the lysate and run on an iodixanol (OptiPrep Sigma D1556) gradient (51). Virus was then concentrated using VivaSpin20 (Sartorius VS20S1) and filter-sterilized (0.45 μm).

### rAAV Characterization

Adeno-associated viral capsid particles were quantified using a discontinuous Laemmli SDS-PAGE setup using standards of Bovine Serum Albumin (BSA) (Pierce Biotechnology/Thermo Scientific 23209) as shown in Kohlbrenner et al. (56). Viral genome size and integrity was determined using alkaline gel electrophoresis (57). Vector genomes were quantified by qPCR. rAAV genome sequences corresponding to a 129 bp fragment (58), of the Cytomegalovirus (CMV) promoter from the transgene and a 62 bp portion of the Inverted Terminal Repeats (ITRs) (59) of the AAV vector were amplified.

### Tissue Culture Infectious Dose (TCID_50_) Determination and Influenza Microneutralization (MN) Assay

Viral diluent consisting of 500 mL DMEM (Sigma D6546), 1% (v/v) Pen-Strep (Sigma P0781), 1% Amphotericin B (v/v) (Sigma A2942), and 1% (v/v) L-glutamine (Sigma G7513) was prepared. For TCID_50_ determination, a 10^–^^4^ dilution of influenza virus was made and diluted ten-fold across the plate. Virus dilutions were transferred to Madin-Darby canine kidney (MDCK) cells at about 70-90% confluency. Plates were incubated for 1 h at room temperature. Dilutions were then discarded and replaced with 100 μL of freshly-prepared Infection Medium [10% (v/v) 10X Minimum Essential Medium (MEM) (Sigma M0275), 0.7% (v/v) Bovine Serum Albumin fraction V solution 7.5% (Sigma 10735078001), 2% (w/v) NaHCO_3_ (Sigma S8761), 1% (v/v) HEPES (Sigma H0887), 0.5% (w/v) DEAE dextran (Sigma 93556), 1% (v/v) Pen-Strep (Sigma P0781), 1% (v/v) L-glutamine (Sigma G7513), 1% (v/v) Amphotericine B (Sigma A2942), 0.7% (v/v) Tosyl phenylalanyl chloromethyl ketone (TPCK)-treated trypsin (Sigma T4376)]. Plates were incubated for 72 h at 37°C and 5% CO_2_. Supernatants were then transferred to round-bottom 96-well plates and 50 μL of 0.7% (v/v) turkey red blood cells (t-RBCs) in PBS were added and the highest dilution with complete hemagglutination in 10 wells in a single row was recorded. The number of hemagglutination positive wells after this row was also recorded and TCID_50_ was calculated using the Reed-Muench method (60).

For the MN assay, 100 μL of either nanobody-Fc (serially diluted two-fold from 256 nM to 128 pM) or heat-inactivated sera from mice treated with AAV encoding transgenes (diluted 1:25 to 1:3200) was added to 100 μL of 10^3^ TCID_50_/mL A/California/07/2009/(H1N1)pdm09. A positive control of ferret antiserum against A/California/07/2009/(H1N1)pdm09 was included. The assay was carried out and neutralization titer was obtained using HA readout; the MN titre was defined as the last well showing complete inhibition of hemagglutination.

### AAV-Nanobody-Fc Tolerability Study

Twenty-eight female BALB/c mice, 6–8 weeks old, were obtained from Charles River Laboratories. Mice were divided into four groups of six for each of the AAV constructs. For every group, the following AAV concentrations in vector genomes (vg) of the rAAV-nanobody Fc: (i) 1.0 × 10^10^, (ii) 3.3 × 10^10^, and (iii) 1.0 × 10^11^ were prepared. Each dose (in 50 μL volume) was given to 2 mice via intramuscular (IM) injection. A control group consisting of 4 mice was given 50 μL PBS. Mice were bled at 0, 2, 4, 6, 8, 10, 12, 16, 20, and 24 weeks post-AAV injection. At the end of 24 weeks, all mice were culled, and terminal bleeds collected.

### *In vivo* Challenge Studies in Mice

Forty female BALB/c 6-8-week-old mice were obtained from Charles River Laboratories. Mice were divided into five groups of eight. On Day 0, mice from groups 1-4 were injected intramuscularly (IM) with a 50 μL volume of 1 × 10^11^ vg AAV-R1a-B6-mIgG1, AAV-R1a-B6-mIgG2a, AAV-R1a-B6, and AAV-cAb1-mIgG2a respectively. Mice from group five were given 50 μL PBS. Thirty-nine (39) days later, mice were bled to determine serum antibody titers. Six weeks (6) after the start of the study, mice were challenged intranasally (IN) with 21 MLD_50_ A/California/07/2009 (H1N1)pdm09 or 10 MLD_50_ A/Vietnam/1194/2004 (H5N1) NIBRG-14ma. Weights and clinical observations were made twice daily for 14 days or until the endpoint of loss of 20% of initial body weight was observed, when lungs were harvested, and terminal bleeds were collected. For the H5N1 challenge experiment, an additional 3 mice per group, were added and culled 3-days post-challenge, to quantify residual influenza in the lungs and for histological analysis.

### Hemagglutination Inhibition Assay (HAI)

All sera were treated with Receptor Destroying Enzyme (RDE) (Seiken) in a 1:4 dilution and incubated overnight at 37°C. Twenty-five microliters of mouse sera was aliquoted into duplicate wells and serially diluted two-fold in PBS before the addition of 4 HAU A/California/07/2009 (H1N1)pdm09 or A/Vietnam/1194/2004 (H5N1) NIBRG-14ma. After 90 minutes incubation at room temperature, 50 μL 0.5% (v/v) T-RBCs was added to each well and HAI titer was determined via the HA readout as described above for the TCID_50_ assay.

### Histology

Mouse lungs were washed in PBS and fixed with 4% paraformaldehyde (PFA). Four-micron-thick sections were then cut using the Leica RM2125 RTS Microtome. Slides were prepared and stained with hematoxylin and eosin (H&E) (Vector Laboratories Inc. H-3502). High definition scans were taken using Pannoramic Digital Slide Scanner (3D HISTECH) and analyzed using CaseViewer 2.3 (3D HISTECH). Inflammation was scored in blinded fashion as follows: 0 = no to minimal inflammation, 1 = occasional infiltration in bronchioles, 2 = infiltration of bronchioles and slight thickening of perivascular walls; 3 = mass infiltration of bronchioles, thickening of perivascular walls, and lung rupture.

### Statistical Analysis

All analyses were performed with GraphPad Prism 8.12 for Windows (GraphPad Software). The Student’s *t* test was used to determine differences between two groups.

## Results

### R1a-B6-Fc Fusions Have Broad Cross-Subtype Specificity and Neutralizing Activity Against Influenza Virus

**FIGURE 1:**
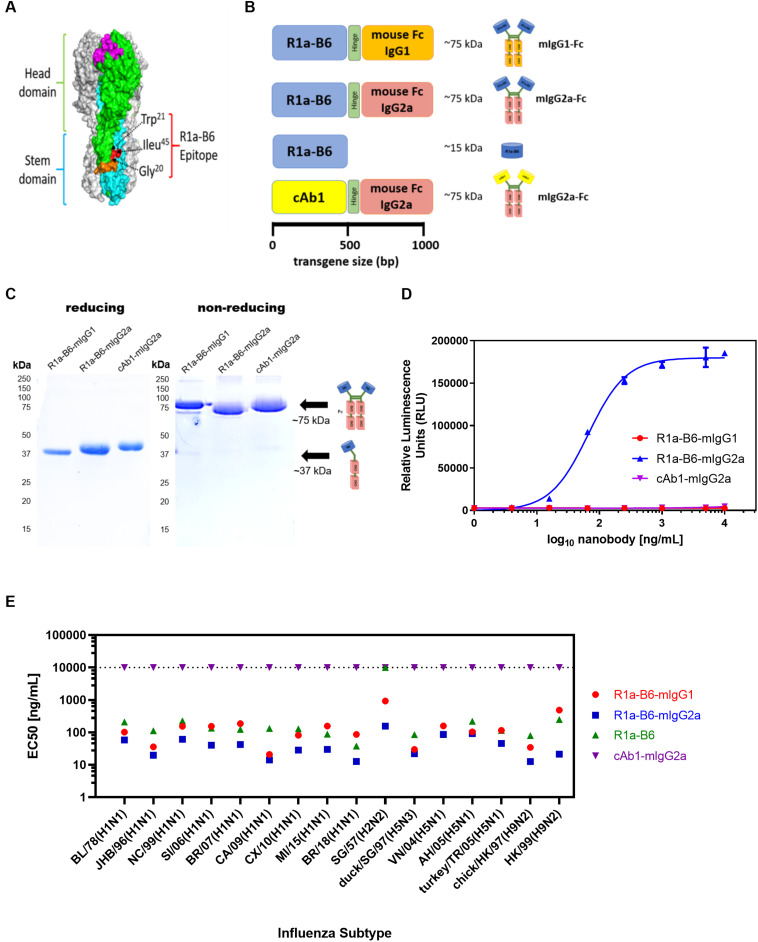
R1a-B6 reformatted for *in vivo* gene delivery. **(A)** Surface structure model of hemagglutinin (HA) trimer of A(H1N1)pdm09 (PDB structure 3AL4) showing the key epitope residues of R1a-B6, Gly20, Trp21, and Ile45 (shown in red) located in the HA stem region (cyan) (40). The receptor binding site (magenta), fusion peptide (orange), and head domain (green) are also illustrated. **(B)** Four constructs, (i) R1a-B6 mouse Fc IgG1, (ii) R1a-B6 mouse Fc IgG2a, (iii) monovalent R1a-B6, and (iv) negative control mouse Fc IgG2a fusion carrying a nanobody, cAb1 [adapted from Arbabi Ghahroudi et al. (54)], specific for chicken egg white lysozyme were produced *in vitro*. These constructs were cloned into an AAV expression system for protein expression *in vivo*. **(C)** Expression and purification of nanobody-Fc fusions. Detection of proteins was carried out under reducing and non-reducing conditions in SDS-PAGE gels. Theoretical molecular weights (MW) for R1a-B6-mIgG1, R1a-B6-mIgG2a, and cAb1-mIgG2a are ∼37 kDa under denaturing (reducing) conditions, and ∼75 kDA under non-reducing conditions. **(D)** *In vitro* ADCC activation. Activation of luciferase reporter gene is shown in relative luminescence units (RLU) as a function of nanobody-Fc concentration. Each well was measured in triplicate. **(E)** Binding of R1a-B6 against a broad range of influenza A subtypes as tested by ELISA. Half maximal effective concentration (EC_50_) was measured in duplicate. There was no binding on A/TX/12(H3N2) or B/Brisbane/08 (data not shown). All values above the dotted line indicate no binding activity.

The cross-neutralizing nanobody R1a-B6 has been shown previously to bind to a highly conserved epitope in the HA stem (40) (Figure 1A), which overlaps the fusion peptide and is predicted to function through inhibiting membrane fusion. To test the prophylactic potential of R1a-B6 (Figure 1A) and the requirement for half-life extension or effector functions we designed four single gene encoding proteins (Figure 1B) by fusion to a mouse Fc fragment of either an IgG1 or IgG2a isotype. Mouse IgG isotypes differentially interact with FcγR on effector cells, with IgG2a being the most potent having high affinity for activating FcγR, whereas IgG1 is the least potent preferentially interacting with inhibitor FcγR (61). After purification, a product of approximately 75 kDa was seen under non-reducing conditions demonstrating the formation of a dimeric Fc fusion protein (Figure 1C). We then tested the ability of R1a-B6-Fc to mediate ADCC via activation of mouse FcγRIV as detected by a luciferase reporter assay; and as expected, only R1a-B6-mIgG2a showed any ADCC activity (Figure 1D). Purified R1a-B6-Fc fusion proteins were then shown to bind to a broad panel of whole influenza virus reference reagents (Figure 1E and Supplementary Figure 1) and neutralize key Group I Influenza A subtypes, A(H1N1), A(H2N2), A(H5N1), and A(H9N2) (Supplementary Table 1). As reported previously (28), converting R1a-B6 into a bivalent format was shown to increase the breadth of neutralizing activity to include the more divergent influenza subtype A(H2N2) rather than increasing maximum levels of potency against A(H1N1) and A(H5N1) (Supplementary Table 1). We have previously speculated that this is related to the mechanism of action of R1a-B6 which mediates its effect after the virus has already attached to the cell surface and been internalized. We have suggested that internalization of the virus nanobody complex may be a rate limiting step in the potency of stem binding antibodies, so conversion to a bivalent format does not increase potency above a certain maximum threshold. As such, converting R1a-B6 from a monovalent to bivalent Fc fusion is seen to enhance potency for more divergent subtypes [i.e., A(H2N2) and A(H9N2)] with a lower affinity interaction, whereas for higher affinity interactions A(H1N1) and A(H5N1), no further enhancement is seen as a maximum level has already been reached.

### *In vivo* Expression of Nanobodies by Intramuscular (IM) AAV Delivery

Upon confirmation of the breadth of reactivity *in vitro* of reformatted R1a-B6, we inserted the corresponding transgenes for R1a-B6-mIgG1, R1a-B6-mIgG2a, R1a-B6, and cAb1-mIgG2a into a recombinant AAV2/8 vector. Transgenes were packaged into AAV using triple transfection (57) and characterized by measuring the number of capsid particles, physical genomes, and gene products via qPCR (data not shown). Capsid and viral genome were found to be intact and of high purity. We then used our AAV viruses for a dose ranging study in mice as described below.

**FIGURE 2:**
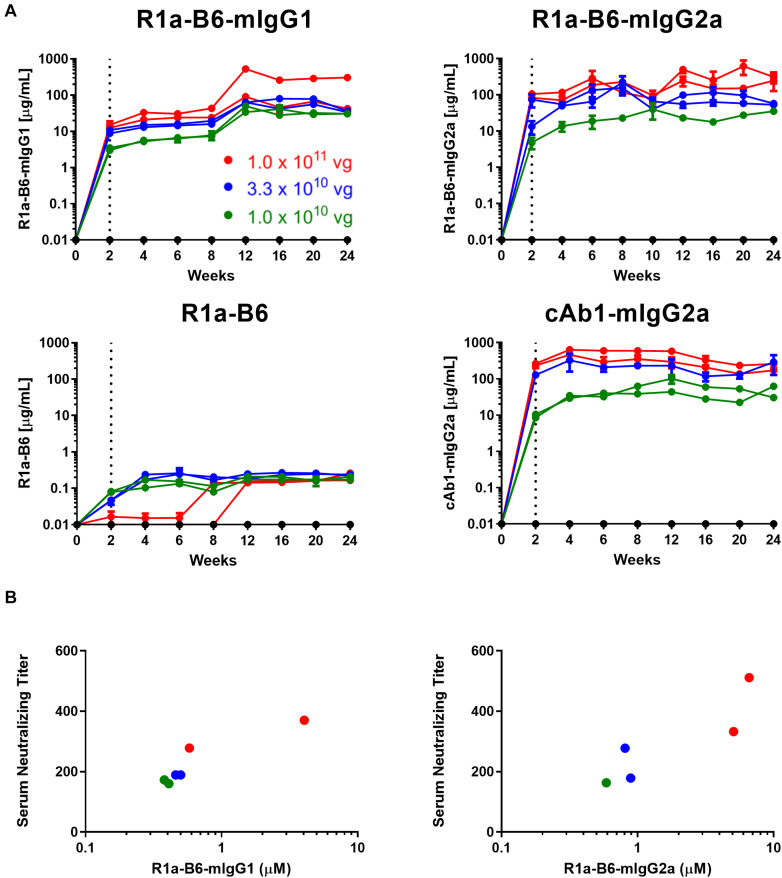
AAV dose ranging study of anti-influenza neutralizing nanobody R1a-B6 in mice. **(A)** Different R1a-B6 constructs and the control nanobody, cAb1, were given via AAV in vector genome (vg) doses of 1.0 × 10^10^ vg (green series), 3.3 × 10^10^ vg (blue series), and 1.0 × 10^11^ vg (red series). Mice that were given PBS are indicated by the black series. Nanobodies in serum of BALB/c mice were measured from week 0 to 24. A mouse given 1.0 × 10^10^ vg of R1a-B6-mIgG2a was culled before the end of the study due to reasons exclusive of AAV delivery. Each individual series corresponds to a single mouse. The dotted line represents the point (2 weeks) at which nanobody expression levels were first detected. **(B)** Neutralizing activity of sera taken from mice 24 weeks after they were injected with different doses of AAV encoding R1a-B6-mIgG1 and R1a-B6-mIgG2a was measured against 10^3^ TCID_50_/mL of CA/09. The serum neutralizing titer is expressed as the reciprocal of the highest dilution at which influenza infection is completely blocked. Mice given R1a-B6 (all doses) did not show any neutralizing activity (data not shown). For all plots, each point represents an individual mouse serum sample, *n* = 2 mice/group.

To test the ability of AAV-mediated delivery to induce muscle specific expression of R1a-B6 transgenes and to determine the optimum AAV dose, a dose ranging study of a single IM injection of 1.0 × 10^11^ vg (vector genomes), 3.3 × 10^10^ vg and 1.0 × 10^10^ vg of each AAV vector was given to BALB/c mice. We tested expression levels in mouse sera for a duration of 24 weeks while observing for any symptoms of stress or discomfort. Detectable levels of nanobody-Fc fusion were seen for all vector doses in mouse sera within 2 weeks of injection (Figure 2A). Nanobody-Fc levels increased, reaching a plateau during weeks 6–12, with concentrations being maintained at a stable level for the duration of the 24-week study with no evidence of reduction. Peak concentrations of ∼560 μg/mL for R1a-B6-mIgG1, ∼1100 μg/mL for R1a-B6-mIgG2a, and ∼600 μg/mL for cAb1-mIgG2a, were detected (Figure 2A). Monovalent R1a-B6 showed very low levels in serum compared to the Fc fusions, reaching a peak concentration of around 0.36 μg/mL by week 6 which remained for the duration of the study. In all cases, mice did not show any symptoms of distress or ill health that could be attributed to AAV or transgene expression for the full 24 weeks.

### *In vitro* Neutralization of A/California/07/2009(H1N1) (CA/09) and Other Strains by Sera From Mice Receiving AAV Transgenes

To determine if mice that received AAV were expressing functional R1a-B6, we performed *in vitro* neutralization assays against pandemic A/California/07/2009(H1N1) (CA/09). Terminal sera (24 weeks) from mice expressing the control nanobody against lysozyme, cAb1, did not show any neutralization of CA/09, as expected (data not shown). Similarly, no neutralization of CA/09 was seen with monovalent R1a-B6, which correlated with serum levels being approximately 1000-fold lower in molar equivalence than the levels attained by the Fc fusions (data not shown). In contrast, sera from mice that received AAV expressing R1a-B6-mIgG1 and R1a-B6-mIgG2a were able to neutralize CA/09 (Figure 2B). The level of neutralization directly correlated with serum concentration as detected by ELISA which in turn correlated with AAV vector genome dosage (Figure 2B). The AAV vector dose of 1.0 × 10^11^ vg gave the highest nanobody serum titer (Figure 2A) and was chosen for protection studies.

**FIGURE 3:**
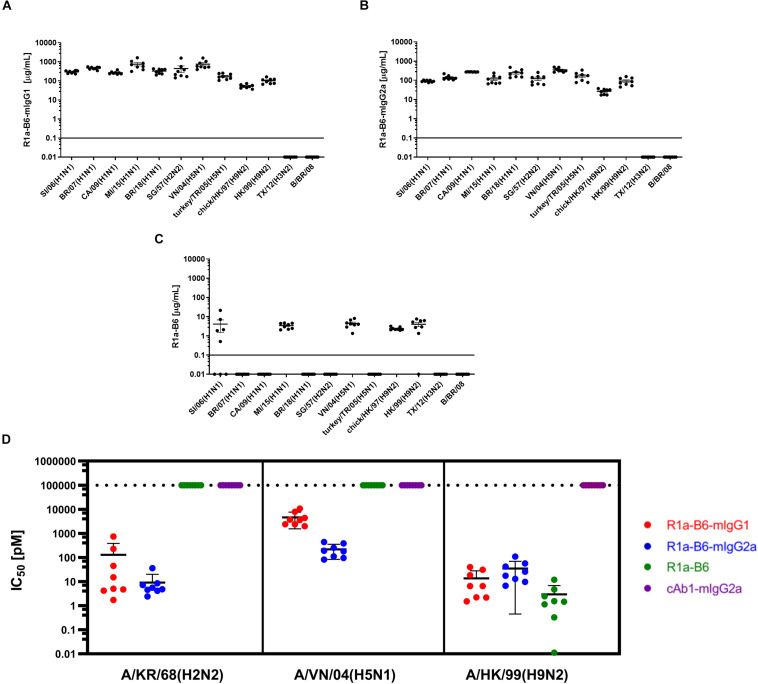
Evaluation of heterosubtypic R1a-B6 reactivity in mouse sera 39 days after IM injection of 1.0 × 10^11^ vg AAV encoding nanobodies. **(A–C)** Binding of R1a-B6 against different influenza subtypes was tested via ELISA. Plates were coated with 5 μg/mL influenza reference reagent (Supplementary Table 3) and the Absorbance at 450 nm was plotted against a serial dilution of recombinant nanobody for standard curve analysis. Recombinant nanobody concentration in serum corresponding to the sample binding activity was plotted. Samples were measured in duplicate. All values below the solid line represent no detectable binding activity. A/Texas/50/2012(H3N2) (TX/12) and B/Brisbane/60/2008 (B/BR/08) served as negative controls. **(D)** *In vitro* neutralization against different influenza pseudotypes was determined via a luciferase-reporter assay and given as IC_50_ values (IC_50_ is half maximal inhibitory concentration). All values above the dotted line indicate no neutralization. For all plots, *n* = 8 mice/group.

We then determined if AAV mediated expression of R1a-B6 present in mouse sera would react with other influenza strains belonging to key influenza subtypes. We obtained mouse sera 6-weeks post AAV IM administration of 1.0 × 10^11^ vg of each R1a-B6 transgene (*n* = 8/group) and tested their cross-subtype specificity and neutralization activity. These mice were naïve and had not been exposed to influenza so immunoreactivity to heterologous strains can be attributed solely to R1a-B6 expressed from the AAV transgene *in vivo* and not to naturally produced antibodies. Sera from mice expressing R1a-B6-mIgG1 (Figure 3A) and R1a-B6-mIgG2a (Figure 3B) were specific against all Group I Influenza A subtypes tested. Mice expressing monovalent R1a-B6 showed lower reactivity against the panel of whole influenza virus antigen standards (Figure 3C), which was consistent with its very low serum levels compared to mice receiving the R1a-B6-Fc fusions. Mice expressing cAb1-mIgG2a and those given PBS did not show any reactivity against influenza virus (data not shown). In addition to cross-subtype specificity, R1a-B6-Fc in mouse sera also demonstrated potent cross-neutralizing ability against Group I Influenza A pseudotypes of subtypes H2, H5, and H9 (Figure 3D). These results demonstrate that R1a-B6-Fc has retained its *in vitro* cross-subtype specificity and neutralizing activity when produced *in vivo*.

### Protection of Mice From Lethal A(H1N1)pdm09 (CA/09) Challenge by AAV-Mediated Delivery of R1a-B6

**FIGURE 4:**
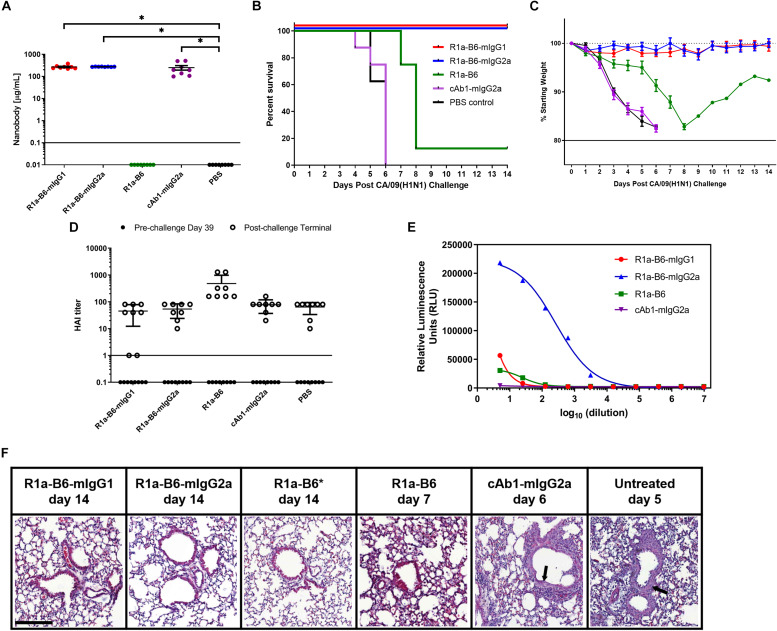
Prophylactic efficacy of AAV expressed nanobodies in a mouse challenge model of pandemic H1N1 CA/09. **(A)** Total nanobody-Fc in serum was measured by ELISA in samples taken 39 days after IM injection of AAV vectors pre-challenge with CA/09. Comparisons are shown in brackets (*p < 0.05). BALB/c mice were then infected intranasally with 21 MLD_50_ (mouse lethal dose) of A/California/07/2009(H1N1)pdm09 42 days post-AAV IM injection. Survival **(B)** and weight loss **(C)** were monitored for 14 days. **(D)** Naturally induced immune response in mice pre- and post-CA/09 challenge was assessed via Hemagglutination Inhibition (HAI) titers 39 days post-AAV administration and 3 days pre-challenge (•), and from post-challenge terminals bleeds (∘). HAI titers are expressed as the highest dilution of serum that inhibited hemagglutination completely. Points below the solid line represent no HAI activity. For panels **(A–D)**, each point represents an individual mouse. Mean and standard error of eight recipient mice per treatment is shown in panels **(A)** and **(C,D)**. **(E)** Binding and activation of mouse FcγRIV effector cells in mice given nanobody-Fc fusions delivered by AAV. Terminal mouse sera were tested for ADCC activity via activation of mFcγRIV from representative mice after IM injection of 1.0 × 10^11^ vg AAV-R1a-B6-mIgG1, AAV-R1a-B6-mIgG2a, AAV-R1a-B6, and AAV-cAb1-mIgG2a. Mice were culled 14 days post-challenge for R1a-B6-mIgG1, R1a-B6-mIgG2a, and R1a-B6, and 6 days post-challenge for cAb1-mIgG2a. Activity is shown in relative luminescence units (RLU) against dilution of mouse sera containing nanobody Fc fusions expressed *in vivo*. Each point is shown as the mean and standard error of three replicates. **(F)** Representative histological lung sections from mice that received AAV encoding R1a-B6-mIgG1, R1a-B6-mIgG2a, R1a-B6, cAb1-mIgG2a, and PBS (untreated) post-infection with CA/09. Lungs were harvested from mice as they exited the study at the days indicated and stained with Hematoxylin and Eosin (H&E). Arrows represent thickening of the alveolar walls. *Represents Mouse 3 (Supplementary Figure 2 and Supplementary Table 2), the only mouse that survived the CA/09 challenge from the R1a-B6 group. Scale bar = 100 μm.

To determine whether AAV delivery of R1a-B6 transgenes protects mice from lethal influenza infection, we injected 1 × 10^11^ vg AAV encoding R1a-B6-mIgG1, R1a-B6-mIgG2a, R1a-B6, and control cAb1-mIgG2a intramuscularly into different groups of BALB/c mice (*n* = 8/group). We also included a negative control group that was given PBS. Thirty-nine days post-AAV injection, we tested for transgene expression prior to lethal challenge with influenza. Mice given R1a-B6-mIgG1, R1a-B6-mIgG2a, and cAb1-mIgG2a have significantly higher concentration in serum than mice given vectors encoding R1a-B6 or PBS (*p* < 0.05) (Figure 4A). In groups receiving nanobody Fc fusions, we detected 300–500 μg/mL levels of nanobody (Figure 4A), whereas for the R1a-B6 transgene without Fc, we were unable to detect any substantial levels in the sera tested.

After confirmation of transgene expression in mouse sera, we proceeded with influenza challenge via intranasal delivery of 21 MLD_50_ (mouse lethal dose) CA/09 42 days after AAV administration. Weight loss and clinical symptoms of influenza were observed for the 14-day study window or until mice exited the study. Animals expressing the control anti-lysozyme specific nanobody cAb1-mIgG2a and those given PBS showed symptoms of influenza including difficulty in breathing, pinched waists and sunken abdomen 3 days post-challenge. These mice lost 20% of weight and were culled by day 6 of the study (Figures 4B,C and Supplementary Figure 2). In contrast, there was no weight loss or symptoms of influenza infection observed in mice given R1a-B6-mIgG1 and R1a-B6-mIgG2a (*n* = 16) for the duration of the study demonstrating complete protection from influenza (Figures 4B,C and Supplementary Figure 2). Mice given monovalent R1a-B6 without mouse Fc, showed symptoms of influenza infection 6 days post-challenge, which was a delay of 3 days compared to control groups given cAb1-mIgG2a and PBS (Figure 4C). Seven mice (*n* = 7) from the monovalent R1a-B6 group eventually succumbed to infection and were culled on days 7–8 due to rapid weight loss. However, a single mouse (Mouse 3) from this group started gaining weight around day 9 and survived until the end of the study (Figures 4B,C, Supplementary Figure 2, and Supplementary Table 2). It was also noted that this mouse was of a higher than average starting weight.

To compare anti-influenza immune response pre- and post-CA/09 challenge we used a hemagglutination inhibition (HAI) assay on terminal mouse serum samples (Figure 4D). HAI is a surrogate test for the presence of neutralizing antibodies which bind to the HA head domain and block the receptor binding site. As expected, pre-challenge samples did not show any HAI activity as the only anti-influenza antibody present was R1a-B6 which has previously been shown to be negative for HAI (28, 40), as is also the case for other HA stem-binding antibodies. Post-H1N1 challenge, mice that received AAV encoding cAb1-mIgG2a and PBS, culled on days 4-6, showed similar HAI titers as groups given AAV encoding R1a-B6, R1a-B6-mIgG1, and R1a-B6-mIgG2a, the latter two groups being mice that survived the influenza challenge with no observable symptoms of infection. We concluded that after influenza challenge, both infection-induced anti-head HA antibodies and anti-stem R1a-B6 nanobodies were present in mouse sera (Figure 4D). These results suggest that naïve mice are still able to mount a natural immune response against CA/09 challenge even after administration of AAV encoding transgenes.

Surprisingly, we did not see any observable difference in the prophylactic efficacy of mice receiving R1a-B6-mIgG2a compared to R1a-B6-mIgG1. This suggested ADCC was not an essential component of the protective mechanism of action of R1a-B6 in this context notwithstanding that the mouse IgG2a isotype version of R1a-B6 was capable of mediating ADCC (Figure 4E).

We also compared the level of inflammation in terminal lung tissue. At the end of the study (14 days post-challenge), mice expressing R1a-B6-mIgG1 and R1a-B6-mIgG2a (*n* = 16) showed clear bronchioles and air sacs with no signs of inflammation (Figure 4F). In contrast, mice expressing cAb1-mIgG2a, 6 mice given R1a-B6, and those given PBS (*n* = 22) demonstrated infiltration of the bronchioles and air sacs with thickening of the perivascular wall (Figure 4F). Two mice from the group given R1a-B6 (Mouse 3 and Mouse 8) did not show any signs of lung inflammation (Figure 4F and Supplementary Table 2). Slight lung infiltration can be seen in lung tissue of mice culled on day 7 given R1a-B6 (Figure 4F).

### Protection of Mice From Lethal A(H5N1) Challenge by AAV-Mediated Delivery of R1a-B6

**FIGURE 5:**
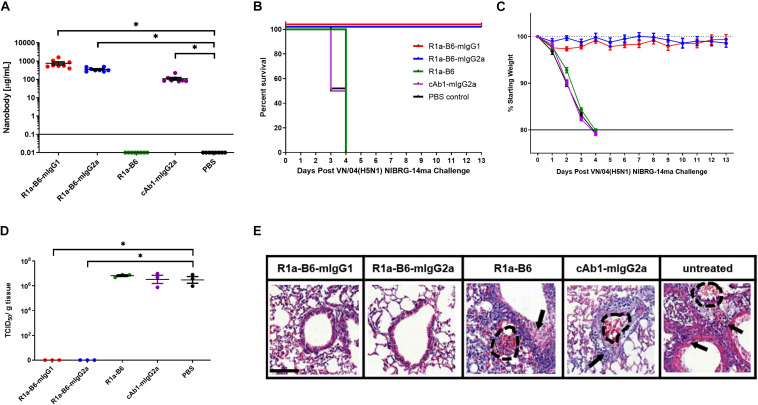
Prophylactic efficacy of AAV expressed nanobodies in a mouse challenge model of VN/04 (H5N1) NIBRG-14ma. **(A)** Total nanobody-Fc in serum was measured by ELISA in samples taken 39 days after IM injection of AAV vectors pre-challenge with VN/04(H5N1). Comparisons are shown in brackets (*p < 0.05). Mice were then infected intranasally with 10 MLD_50_ (mouse lethal dose) of A/Vietnam/1194/2004 (H5N1) NIBRG-14ma 42 days post-AAV IM injection. Survival **(B)** and weight loss **(C)** were monitored for 13 days. **(D)** Residual viral load was determined as indicated by TCID_50_/g of tissue from lung homogenates 3 days post-challenge with 10 MLD_50_ VN/04. Each dot represents an individual mouse. Comparisons are shown in brackets (*p* < 0.05). **(E)** Representative histological lung sections from mice that received AAV encoding R1a-B6-mIgG1, R1a-B6-mIgG2a, R1a-B6, cAb1-mIgG2a, and PBS (untreated) 3 days post-infection with VN/04 NIBRG-14ma. Lungs were harvested from mice and stained with Hematoxylin and Eosin (H&E). Arrows represent thickening of the alveolar walls and lung ruptures are represented by dashed lines. Scale bar = 100 μm.

To assess if the prophylactic efficacy of R1a-B6 can extend to a different influenza subtype, we tested in an H5N1 influenza challenge model. The study design was the same as that of the CA/09 challenge except that mice received 10 MLD_50_ of NIBRG-14ma (VN/04), a mouse-adapted strain of a re-assortant virus derived from A/Vietnam/1194/04 (H5N1). Symptoms of influenza and rapid weight loss were observed 2-days post-challenge in mice given AAV encoding cAb1-mIgG2a, PBS and monovalent R1a-B6, which was earlier than those observed after the CA/09 challenge experiment (Figure 5C and Supplementary Figure 3). All mice from these groups (*n* = 24) were culled by day 4 (Figure 5B). Similar to the CA/09 challenge, all mice given R1a-B6-mIgG1 and R1a-B6-mIgG2a (*n* = 16) did not show any symptoms of influenza or any appreciable weight loss for the 13 days of the study (Figure 5C), demonstrating the cross-subtype prophylactic efficacy of the R1a-B6-Fc fusions. We were also unable to see any difference between the two different isotype variants of R1a-B6 as both provided complete protection. However, unlike the CA/09 challenge, we did not see any delay in the onset of infection in the group that received R1a-B6 (Figure 5B) suggesting that the level of R1a-B6 sustained in mouse sera (Figure 5A) was not sufficient to protect mice against this strain.

To further characterize the extent of protection against NIBRG-14ma (VN/04) we culled 3 mice per group 3-days post-challenge in addition to the 8 mice per group as they reached the mandated study endpoint to evaluate residual virus in the lungs. Mice given R1a-B6-mIgG1 and R1a-B6-mIgG2a have significantly lower TCID_50_/g of VN/04 than mice given R1a-B6, cAb1-mIgG2a and PBS (*^∗^p* < 0.05) (Figure 5D). Specifically, no virus was detected in the lungs of mice receiving R1a-B6-Fc fusions 3 days post-challenge whereas virus could be detected in groups given R1a-B6, cAb1-mIgG2a, and PBS (Figure 5D). The mice (*n* = 9) had average viral titers of 10^6.^^8^ TCID_50_/g of NIBRG-14ma (Figure 5D). In contrast to the CA/09 challenge study, we did not see any HAI activity against H5N1 either pre- or post-challenge in any of the groups (data not shown). We speculate that the absence of head specific antibodies, which would be a surrogate indication of a natural immune response to influenza, is due to mice needing to be culled earlier, 3–4 days post-infection, which is insufficient time for mice to mount an immune response. For mice receiving the R1a-B6-Fc fusions, we speculate that the virus was cleared before any immune response could be elicited, which is supported by the complete absence of detectable virus 3 days post challenge (Figure 5D).

We also scored the level of inflammation in lung tissue via histological staining from the three mice we culled per group 3 days post-challenge. At 3-days post-challenge, lungs taken from mice expressing R1a-B6-mIgG1 and R1a-B6-mIgG2a (*n* = 6) showed no signs of inflammation with clear bronchioles and air sacs which is consistent with them having no viral load (Figure 5E), indicating full protection from influenza infection. In contrast, mice expressing R1a-B6, cAb1-mIgG2a, and those given PBS (*n* = 9) demonstrated extensive infiltration of the bronchioles and air sacs, thickening of the perivascular wall, and lung ruptures as seen in the accumulation of red blood cells (RBCs) in the alveolar space (Figure 5E).

## Discussion

Vaccination is the mainstay of influenza infection control, however, high-risk patient groups such as the elderly and immune-compromised, do not respond well to vaccines (2, 5, 62). As such, there is a need for additional prophylactic and therapeutic approaches that are independent of the patient’s immune system and the prior availability of the matching influenza strain (63, 64). One approach, which is of considerable interest, is passive immunotherapy or prophylaxis with broadly neutralizing monoclonal antibodies which bind to conserved epitopes on influenza HA (10, 13, 20, 65–67). We have previously described R1a-B6 which has an epitope overlapping the fusion peptide, suggesting a post-viral attachment mechanism of action through the inhibition of viral membrane fusion (28). In this study, we have evaluated the prophylactic efficacy of R1a-B6 using intramuscular AAV delivery. We have chosen to explore intramuscular delivery instead of intranasal delivery as others have chosen (22, 23, 68) as this has been reported to produce higher and more durable expression levels in systemic circulation (20, 50). For example in macaques, AAV expression in the nasal cavity was shown to decrease after 3–4 months which was likely due to the turnover of nasal epithelial cells and loss of transgene expression (22). In contrast, the low turnover rate of muscle cells should, in principle, mean that the AAV transgenes can be maintained for much longer (52, 53). This delivery method is also simpler to implement in resource poor settings alongside other immunizations.

We have expressed R1a-B6 both as a single domain and as an Fc fusion of IgG1 and IgG2a isotypes to evaluate the importance of half-life extension and effector function for *in vivo* efficacy. We have shown that a single IM injection gave robust expression of R1a-B6-Fc fusions (0.5–1.1 mg/mL) in the systemic circulation which was sustained for a minimum of 6 months with no observable ill effects (Figure 2A). We did not see any evidence of reduction over time which suggests that there was no significant loss of transgenes, transfected cells or immune mediated clearance of R1a-B6 or AAV vector. The level of production is higher than previously reported for conventional human mAbs delivered intramuscularly (50-200 μg/mL) (20) or intranasally (1 μg/mL) (22, 23). In one case, much lower expression levels were reported for intranasal delivery of 0.5 μg/mL in nose, 2 μg/mL in lung, and 120 ng/mL in circulation which decline over time (22). The very high serum concentration we see in our study might reflect the simpler nanobody transgene as compared to a conventional monoclonal antibody which requires the stable assembly of two light chains and two heavy chains inside the cell for successful secretion. It will be interesting to directly compare the impact of route of vector delivery on antibody efficacy as at present, it is unclear if localized AAV gene delivery in the nasal passages will be able to provide long term durable expression.

We have shown complete protection of mice by R1a-B6-Fc fusion in challenge models covering two different influenza subtypes of pandemic potential. The mice that survived had no residual virus in their lungs and normal lung morphology with complete viral clearance. We were also able to see an HAI response post challenge with A(H1N1)pdm09, suggesting that AAV delivery and expression of R1a-B6 which neutralizes virus by post attachment mechanisms and is itself HAI negative did not interfere with the natural immune response to virus. Surprisingly, monovalent R1a-B6, which was only detectable at very low levels in circulation (Figure 4A), was able to delay the onset of infection by at least 3 days after a lethal challenge with H1N1 (Figure 4C), with one mouse surviving until the end of the study. We can infer that continuous AAV production of R1a-B6 partially offsets the rate of clearance by glomerular filtration in the kidneys, providing some protection even in the absence of Fc mediated half-life extension. However, fusion of R1a-B6 to an Fc fragment dramatically improved its pharmacokinetic and pharmacodynamic properties allowing protective levels to be reached over an extended period. The mechanism is expected to be due to FcRn-mediated recycling or Fc-mediated distribution and retention in tissues (44).

Monoclonal antibodies which bind to the HA stem have been described as utilizing additional mechanisms to neutralize influenza virus *in vivo* through recruitment of the effector arm of the immune system via Fc-Fcγ receptor interaction (45, 46). For instance, HA stem-binding antibodies have been reported to neutralize influenza virus by binding HA on virally infected cells and recruiting NK cells to mediate ADCC (69, 70). As R1a-B6 is functionally equivalent to other HA stem binding human mAbs (10, 13), it may also have the potential to utilize these additional mechanisms of action. To evaluate the extent ADCC might contribute to the *in vivo* efficacy of R1a-B6 we re-formatted it as either a mouse IgG2a-Fc fusion (ADCC+) or a mouse IgG1-Fc fusion (ADCC-) to ensure compatibility with mouse FcγRs as confirmed *in vitro* (Figure 1D). As protection was complete with R1a-B6 formatted with either isotype we were not able to see any obvious difference in efficacy between R1a-B6-Fc fusions *in vitro* (Figure 1E and Supplementary Table 1) or *in vivo* (Figures 4B, 5B). This contrasts with previous studies using passive transfer of the stem binding human mAb F16 which was similarly tested as a mouse IgG1 and IgG2a in the context of the mouse FcγR system. In this study, only mice that received F16-mouse-IgG2a showed 100% survival from lethal PR8 (H1N1) challenge whereas mice that received F16-mouse-IgG1 did not survive (46). However, further studies by the same group showed that the requirement for FcγR interactions and ADCC was dose dependent with the inference being that efficacy through passive transfer of high doses of FI6 was FcγR interaction independent (45). We speculate, as we have been able to generate very high stable concentrations in serum using intramuscular AAV delivery of R1a-B6-mIgG2a and R1a-B6-mIgG1, that viral neutralization via inhibiting viral membrane fusion is sufficient to provide protection with no need for FcγR interactions. In addition, the observation of a protective effect of monovalent R1a-B6, albeit limited, is an unexpected finding and again suggests continual expression *in vivo* may to some extent offset the need for Fc effector functions.

This independence from the host immune system is an attractive facet to include when considering the optimal antibody format for long-term expression *in vivo* against a variable pathogen. Eliminating the need for an Fc mitigates some of the concerns of antibody dependent enhancement of influenza (71–73) mediated by interactions with the effector arm of the immune system or pro-inflammatory functions that can lead to cytokine release and other toxic side effects (74). In addition, this may reduce the risk of transgene immunogenicity through Fc mediated uptake and antigen presentation by immune cells (70, 75). The hypothesis that the effector arm of the immune system is not essential for R1a-B6 to provide sufficient protection against influenza opens up new opportunities for designing an optimized transgene using alternative approaches to half-life extension (76).

In this study we have shown that the use of AAV vectors to deliver R1a-B6 through a single IM injection is an attractive approach to confer broad-based protection against important influenza subtypes (77) which can enhance preparedness against future potential influenza pandemics. In addition, our data suggest that the potency of R1a-B6 does not depend on the coupling of Fc effector functions to HA binding for neutralization of influenza. Highly pathogenic avian influenza H5N1, as well as viruses of subtype H2N2 and H1N1 continue to represent pandemic threats and while pre-pandemic vaccines against these strains have been stockpiled by some governments, there is uncertainty as to exactly what strain could emerge or if these vaccines would be sufficiently antigenically matched to be effective particularly in at risk patient groups. Our alternative approach mitigates these risks and combines the advantages of AAV mediated gene therapy with highly potent cross-neutralizing nanobodies against influenza to provide broad protection independent of the prior availability of the influenza strain and the need for a natural host induced immune response.

## Data Availability Statement

The raw data supporting the conclusions of this article will be made available by the authors, without undue reservation, to any qualified researcher.

## Ethics Statement

The animal studies using AAV and influenza challenge were reviewed and approved under Home Office Licenses: PPL70/8091, P856F6831, and P30D4C513.

## Author Contributions

JD, MS, MC, OE, YT, and SH conceived and designed the experiments and analyzed the data. JD, MS, PR, and KZ performed the experiments. NT contributed the reagents. JD, YT, and SH drafted the manuscript. OE, MC, and MS revised the manuscript.

## Conflict of Interest

The authors declare that the research was conducted in the absence of any commercial or financial relationships that could be construed as a potential conflict of interest.
