# Supplementary material for: Protection From Influenza by Intramuscular Gene Vector Delivery of a Broadly Neutralizing Nanobody Does Not Depend on Antibody Dependent Cellular Cytotoxicity
Source: Front Immunol. 2020 May 29;11:627. doi: 10.3389/fimmu.2020.00627 (PMC7273724; doi:10.3389/fimmu.2020.00627)
Supplement: Supplementary file 1 [file Data_Sheet_1.docx]

Supplementary Material


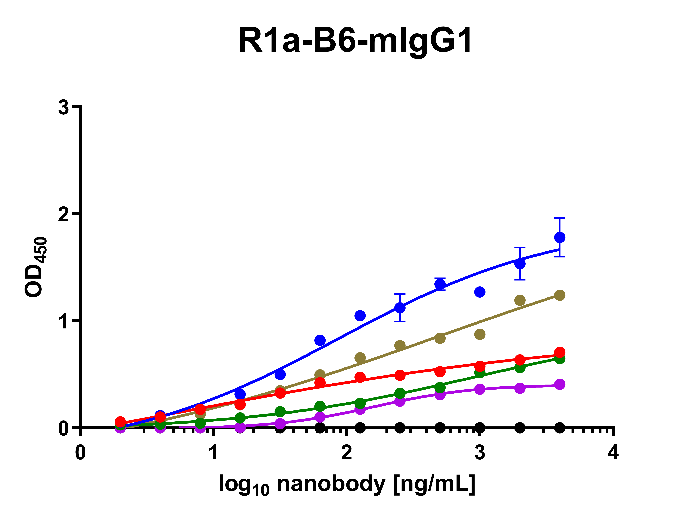

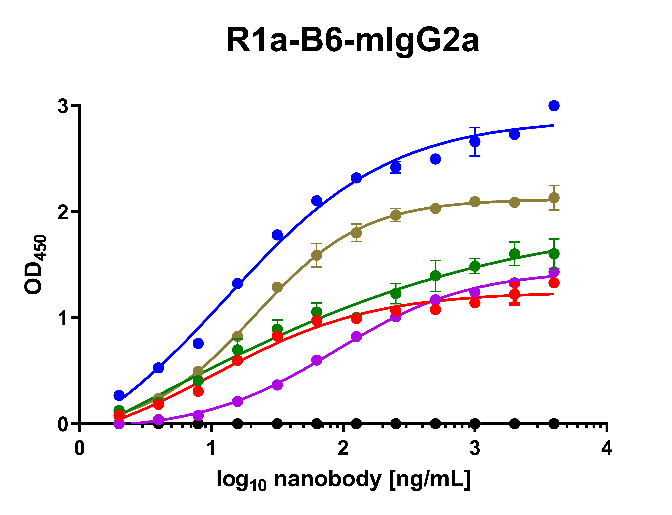

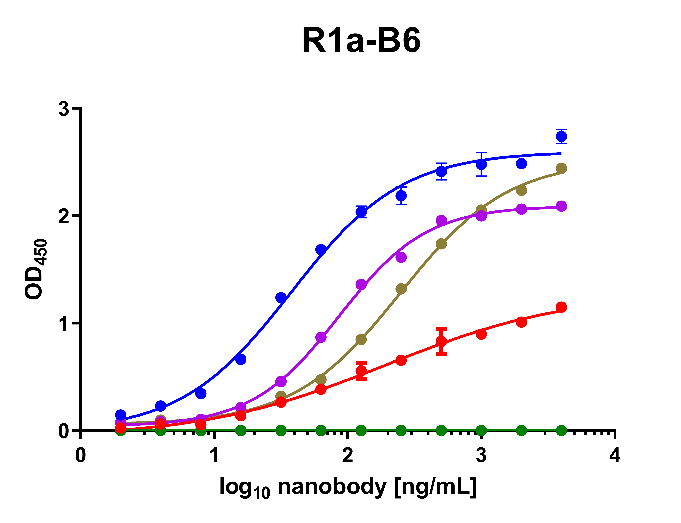

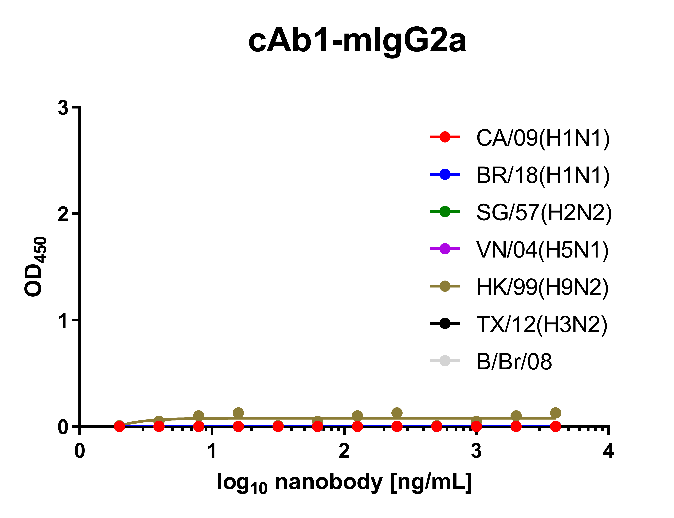


**Supplementary Figure 1.** ELISA showing binding of R1a-B6 to different influenza strains. The Optical Density (OD) at 450 nm was plotted against a serial dilution of nanobody from 4 µg/mL to 2 ng/mL. Binding was measured in duplicate. CA/09(H1N1) - red series, BR/18(H1N1) – blue series, SG/57(H2N2) – green series, VN/04(H5N1) – violet series, HK/99(H9N2) – olive series, TX/12(H3N2) – black series, B/Br/08 – gray series.


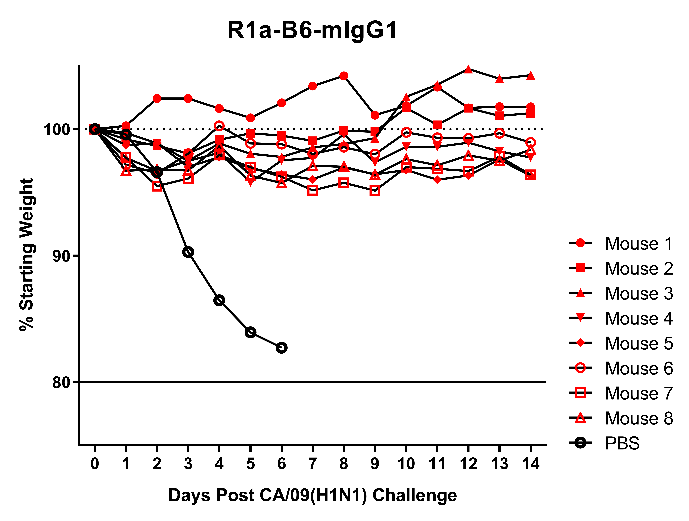

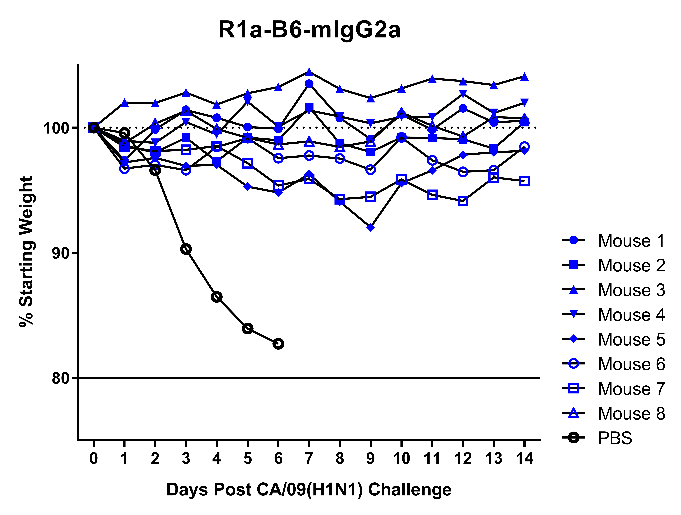


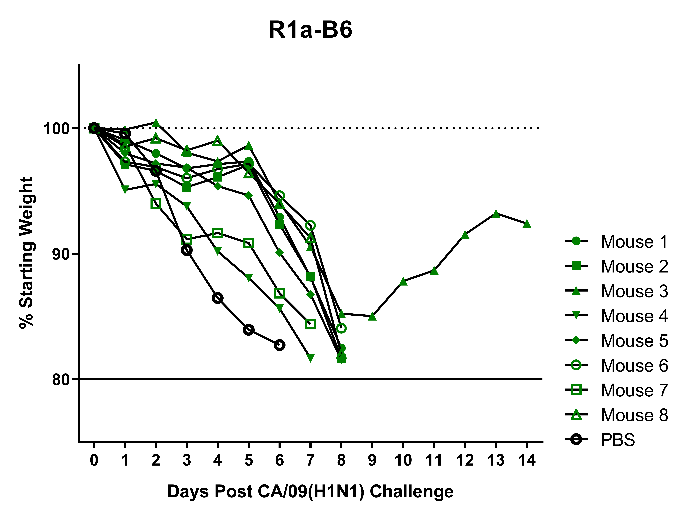

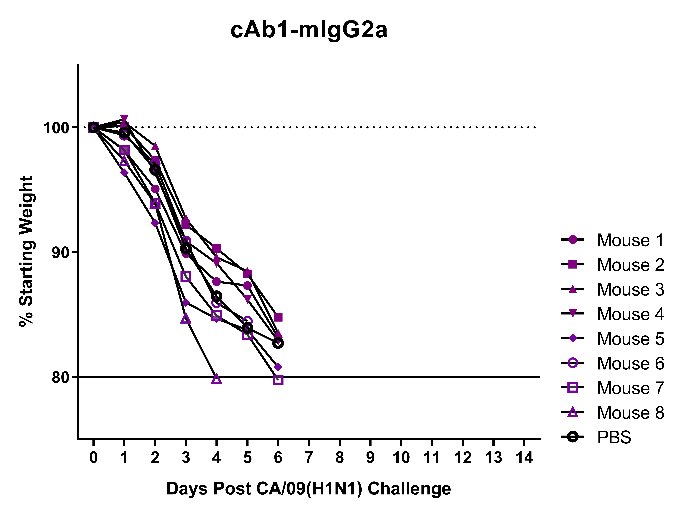


**Supplementary Figure 2.** Evaluation of protection conferred by R1a-B6 from lethal pandemic H1N1 influenza infection. BALB/c mice were administered with AAV-encoding nanobodies six weeks prior and were infected intranasally with 21 MLD_50_ CA/09 influenza. Weight loss and signs and symptoms of influenza were monitored for 14 days in recipients of AAV encoding R1a-B6-mIgG1, R1a-B6-mIgG2a, R1a-B6, and cAb1-mIgG2a. The plots show mean and standard error of eight recipient mice per nanobody construct. Mice were culled when they reached the mandated endpoint of the study, loss of 20% body weight, or if they were observed to suffer from severe distress and/or symptoms of influenza. PBS series is plotted as the mean weight of 8 mice.


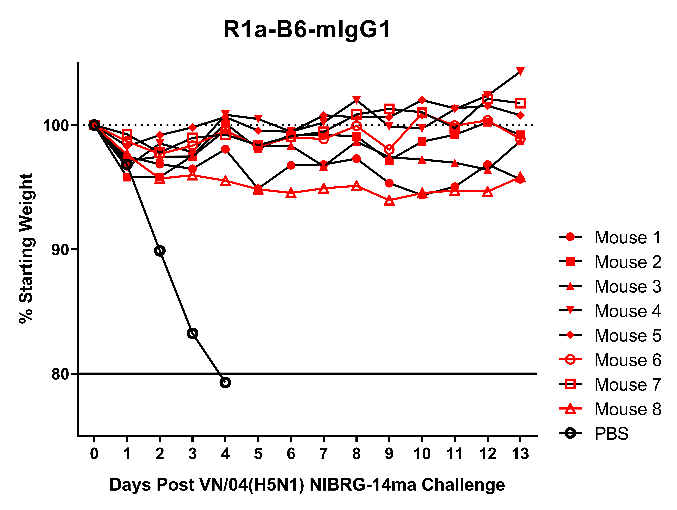

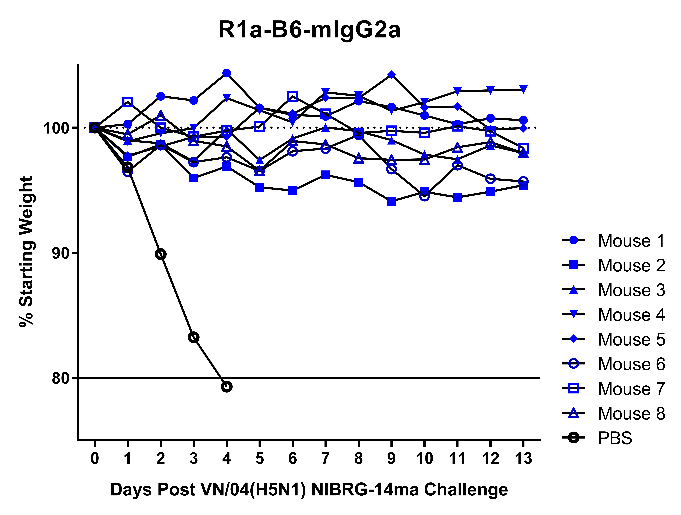


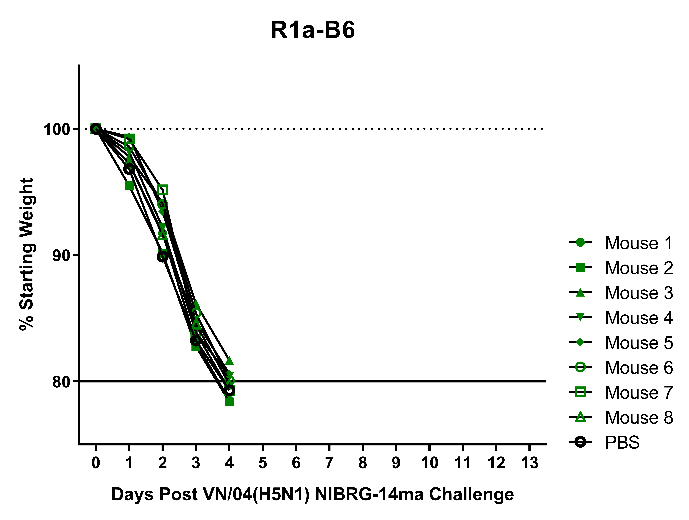

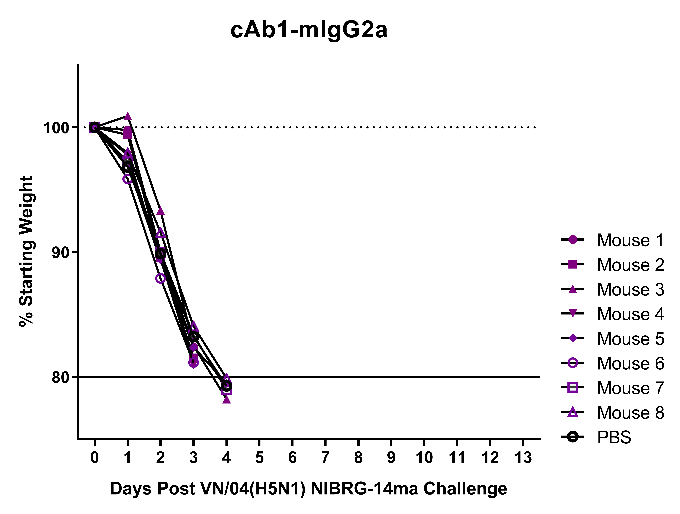


**Supplementary Figure 3.** Evaluation of protection conferred by R1a-B6 from lethal highly pathogenic avian influenza H5N1 influenza infection. BALB/c mice were administered with AAV-encoding nanobodies six weeks prior and were infected intranasally with 10 MLD_50_ VN/04 NIBRG-14ma influenza. Weight loss and signs and symptoms of influenza were monitored for 13 days in recipients of AAV encoding R1a-B6-IgG1, R1a-B6-IgG2a, R1a-B6-HisMyc, and cAb1-IgG2a. The plots show mean and standard error of eight recipient mice per nanobody construct. Mice were culled when they reached the mandated endpoint of the study, loss of 20% body weight, or if they were observed to suffer from severe distress and/or symptoms of influenza. PBS series is plotted as the mean weight of 8 mice.

**Supplementary Table 1.** Nanobody-Fc neutralization of different influenza pseudotypes *in vitro*.

| **Influenza**  **Virus Subtype** | **IC_50_ (nM)** | | | |
| --- | --- | --- | --- | --- |
|  | **R1a-B6-**  **mIgG1** | **R1a-B6-mIgG2a** | **R1a-B6** | **cAb1-**  **mIgG2a** |
| **A/CA/09(H1N1)*** | 5.20 ± 0.01 | 7.99 ± 0.01 | 4.27 ± 0.01 | - |
| **A/KR/68(H2N2)** | 0.146 ± 0.040 | 0.110 ± 0.006 | - | - |
| **A/VN/04(H5N1)** | 3.621 ± 0.030 | 2.431 ± 0.014 | 0.650 ± 0.002 | - |
| **A/HK/99(H9N2)** | 0.027 ± 0.002 | 0.082 ± 0.001 | - | - |

- A/CA/09(H1N1) neutralization assay was carried out using a standard cell-based assay with hemagglutination readout and using live virus. All other assays were carried out using a Luciferase reporter assay and influenza pseudotype viruses (*n=3*). IC_50_ is half maximal inhibitory concentration. ( - ) indicates no neutralization activity.

**Supplementary Table 2.** Summary of relevant values and clinical observations of mice with unusual results that were given R1a-B6 via IM AAV delivery and challenged with CA/09.

| **Mouse Number** | **Day of start of symptoms** | **Clinical Symptoms** | **Day culled** | **TCID_50_/g tissue** | **HAI TITER (pre-challenge)** | **HAI TITER (terminal)** | **Serum Neutralizing Titer** | ***Degree of Inflammation (0-3)** |
| --- | --- | --- | --- | --- | --- | --- | --- | --- |
| R1a-B6 Mouse 3 | 6 | Poorly appearance from day 6, difficulty in breathing from days 7-9, abdominal contractions day 7-8, lost 15% initial body weight by day 7, started gaining weight day 10 | 14 (end of study) | 0 | 0 | 320.00 | 80 | 0 |
| R1a-B6 Mouse 8 | 6 | Poorly appearance from day 6, difficulty in breathing from days 7-8, abdominal contractions day 7-8, lost 20% initial body weight by day 8 and was culled | 8 | 0 | 0 | 320.00 | 160 | 0 |

***** Degree of inflammation is scored from 0-3 with 3 showing the highest degree of inflammation as observed via H&E staining. Lung sections were taken on the day of culling (terminal).

**Supplementary Table 3.** List of influenza antigen standards from the National Institute for Biological Standards and Control (NIBSC) used in this study.

| **Influenza Antigen Standard** | **NIBSC code** | **Abbreviation** |
| --- | --- | --- |
| A/Brazil/11/78(H1N1) | 79/560 | BL/78(H1N1) |
| A/Johannesburg/82/96(H1N1) NIB-39 | 97/518 | JHB/96(H1N1) |
| A/Beijing/262/95(H1N1) NYMC X-127 | 97/760 | BX/95(H1N1) |
| A/New Caledonia/20/99(H1N1) IVR-116 | 06/170 | NC/99(H1N1) |
| A/Solomon Islands/3/2006(H1N1) IVR-145 | 07/102 | SI/06(H1N1) |
| A/Brisbane/59/2007(H1N1) IVR-148 | 08/100 | BR/07(H1N1) |
| A/California/07/2009(H1N1)pdm09 NYMC X-179A | 09/174 | CA/09(H1N1) |
| A/Michigan/45/2015(H1N1) NYMC X-275 | 17/182 | MI/15(H1N1) |
| A/Brisbane/02/2018(H1N1) IVR-190 | 18/238 | BR/18(H1N1) |
| A/Singapore/1/57(H2N2) | 99/714 | SG/57(H2N2) |
| A/Uruguay/716/2007(H3N2) NYMC X-175C | 08/278 | UY/07(H3N2) |
| A/Texas/50/2012(H3N2) NYMC X-223A | 13/116 | TX/12(H3N2) |
| A/Vietnam/1194/2004(H5N1) NIBRG-14 | 09/184 | VN/04(H5N1) |
| A/Anhui/01/2005(H5N1) BCD-RG6 | 07/290 | AH/05(H5N1) |
| A/turkey/ Turkey/01/2005(H5N1) | 07/112 | turkey/TR/05(H5N1) |
| A/duck/Singapore/Q/F119-3/1997(H5N3) NIB-40 | 00/552 | duck/SG/97(H5N3) |
| A/chick/Hong Kong/G9/97(H9N2) NIBRG-91 | 08/228 | chick/HK/97(H9N2) |
| A/Hong Kong/1073/99(H9N2) | 08/208 | HK/99(H9N2) |
| A/Brisbane/10/2010(H1N1) cell derived antigen | 11/134 | BR/10(H1N1) |
| A/Christchurch/16/2010(H1N1) NIB-74 | 10/258 | CX/10(H1N1) |
| B/Brisbane/60/2008 | 13/234 | B/BR/08 |
